# A novel quantification method for automatic computation of breast density from mammography images using deep learning

**DOI:** 10.1007/s12282-025-01808-1

**Published:** 2026-01-09

**Authors:** Kenichi Inoue, Aika Kawasaki, Takeshi Sasaki, Takako Doi

**Affiliations:** 1 Breast Cancer Center, Shonan Memorial Hospital, 2-2-60 Fueda, Kamakura, Kanagawa 248-0027 Japan; 2 Keio Clinic for Diagnostic Digital pathology, Center for Cancer Genomics, Keio University School of Medicine, 35 Shinano-machi, Shinjuku-ku, Tokyo, 160-8582 Japan

**Keywords:** Breast cancer, Dense breast, Breast density, Mammography, Artificial intelligence

## Abstract

**Purpose:**

To develop and validate a fully automated deep-learning pipeline that quantifies mammographic dense rate (MDR) on processed mammograms, and to characterize age-related MDR trajectories in large-scale screening cohort.

**Materials and methods:**

Segmentation AI was built with a U-Net segmentation network. The AI model was trained with 300 mediolateral-oblique images (240 for training, 60 for testing) in which pectoral muscle, glandular and fatty tissue were manually annotated. Segmentation accuracy was evaluated with the DICE coefficient whether AI could extract the segment of gland, pectoral muscle and fatty tissue. MDR was calculated, using the AI model. MDR was defined as the proportion of glandular pixels whose intensity exceeded the mean intensity of the segmented pectoral muscle. For each 240,465 processed MLO images from 134,411 women aged 40–79 years acquired in the Yokohama municipal cancer-screening program between January 2019 and April 2022, MDR was calculated.

**Results:**

The segmentation model achieved DICE coefficient of 0.967. Mean age was 56.6 ± 11.2 years. Mean MDR was 31.7 ± 25.9% and the median MDR was 26.9%. MDR declined steeply from the early 40s to the late 50s, then plateaued. Longitudinal analysis revealed two distinct patterns; a rapidly decreasing group and a persistently high-density group. Increased MDR may serve as an imaging biomarker preceding cancer diagnosis.

**Conclusion:**

The proposed vendor-agnostic U-Net pipeline enables accurate, standardized MDR measurement on processed images of mammograms, eliminating observer variability. Large-scale deployment elucidated granular age-specific density dynamics and supports quantitative MDR as a practical tool for risk-adapted breast-cancer screening and personalized selection of adjunct imaging modalities.

## Introduction

Dense breast tissue is an independent risk factor for breast cancer and can obscure malignancies on mammography [1]. The American College of Radiology’s Breast Imaging Reporting and Data System (BI-RADS) classifies breast composition into four categories: fatty, scattered fibroglandular, heterogeneously dense, and extremely dense [2]. This standardized classification system plays a crucial role in the early detection of malignancies [3]. In Japan, breast composition is assessed based on the Breast Composition Judgment Atlas [4], developed by the Japan Central Committee on Quality Assurance for Mammography. However, this assessment relies on visual inspection, which inevitably makes variability in judgment. This discrepancy in assessing by readers results in unreliability due to lack of uniformity. On the other hand, advancements in artificial intelligence (AI) are rapidly transforming this field. Recent progress in AI has enabled more accurate and consistent evaluations of dense breast tissue, offering new possibilities for early breast cancer detection. Our research has focused on quantitatively assessing breast density automatically using AI, rather than qualitative evaluations. Through this approach, we have achieved a certain level of consistency. Based on these findings, we have also examined changes in breast density associated with aging. An automated quantification system using AI provides a standardized approach, minimizing human bias and error.

## Materials and methods

**Fig. 1:**
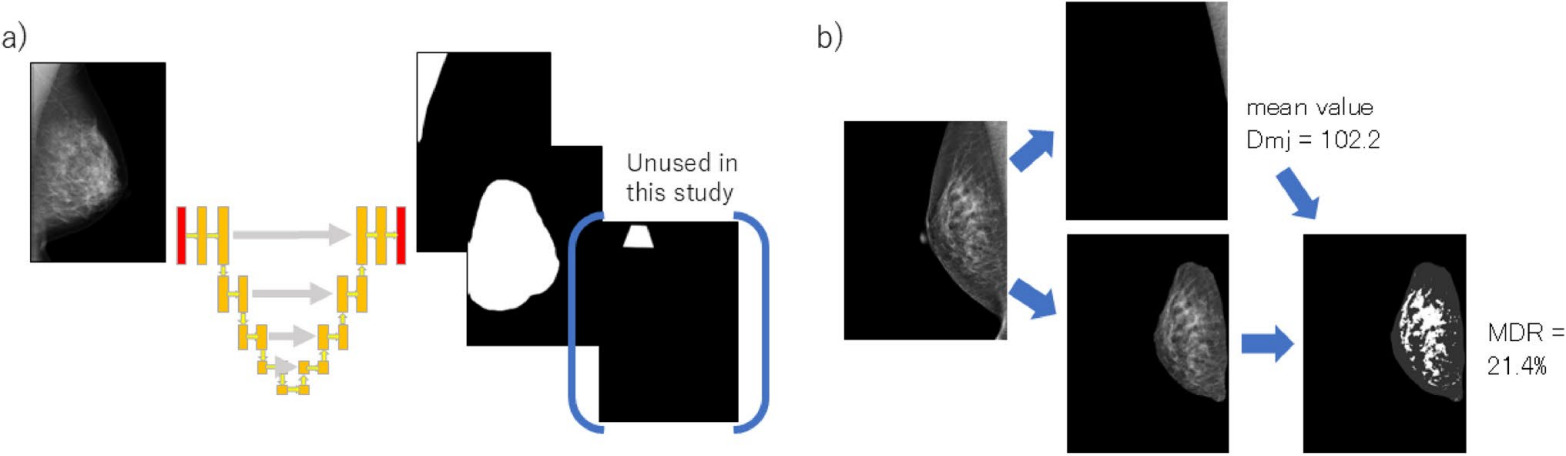
U-Net architecture. **a**) When a mammography image is input, it outputs segmented mask images for glandular tissue, the pectoralis major muscle, and fat. **b**) From the mammography image and its mask image, the pectoralis major muscle is extracted and its mean pixel value is calculated (D_mj_). Then glandular tissue is extracted and the percentage of dense pixel greater than D_mj_ is calculated (MDR)

**Fig. 2:**
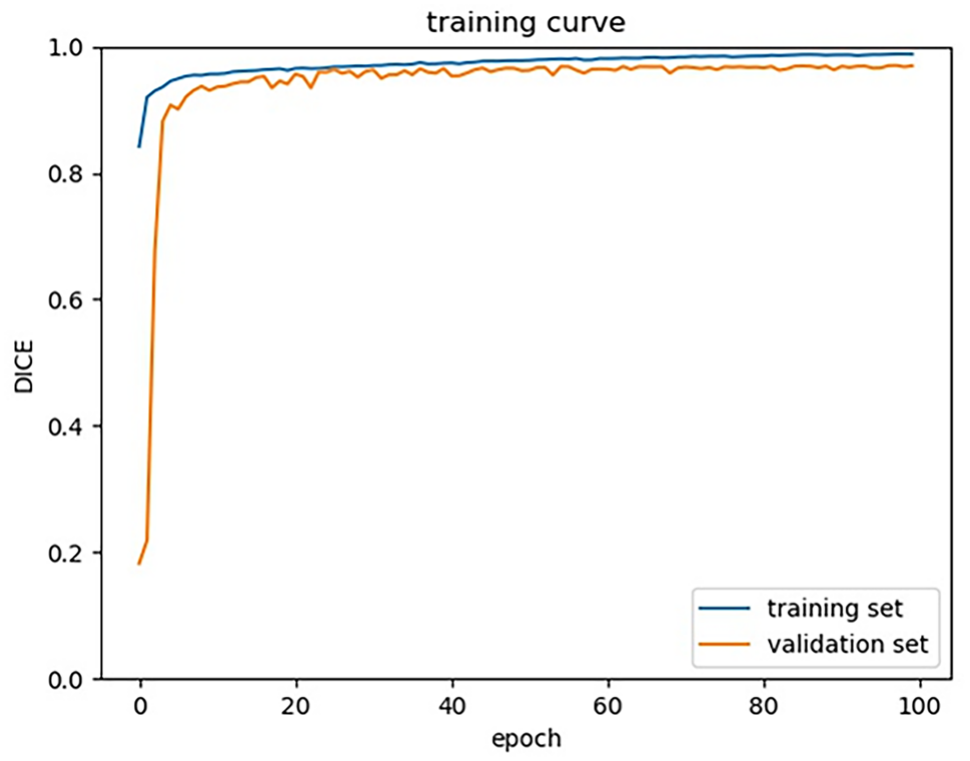
DICE coefficient. U-Net is properly trained and its accuracy is reached at the plateau after 100 epoch of training

**Fig. 3:**
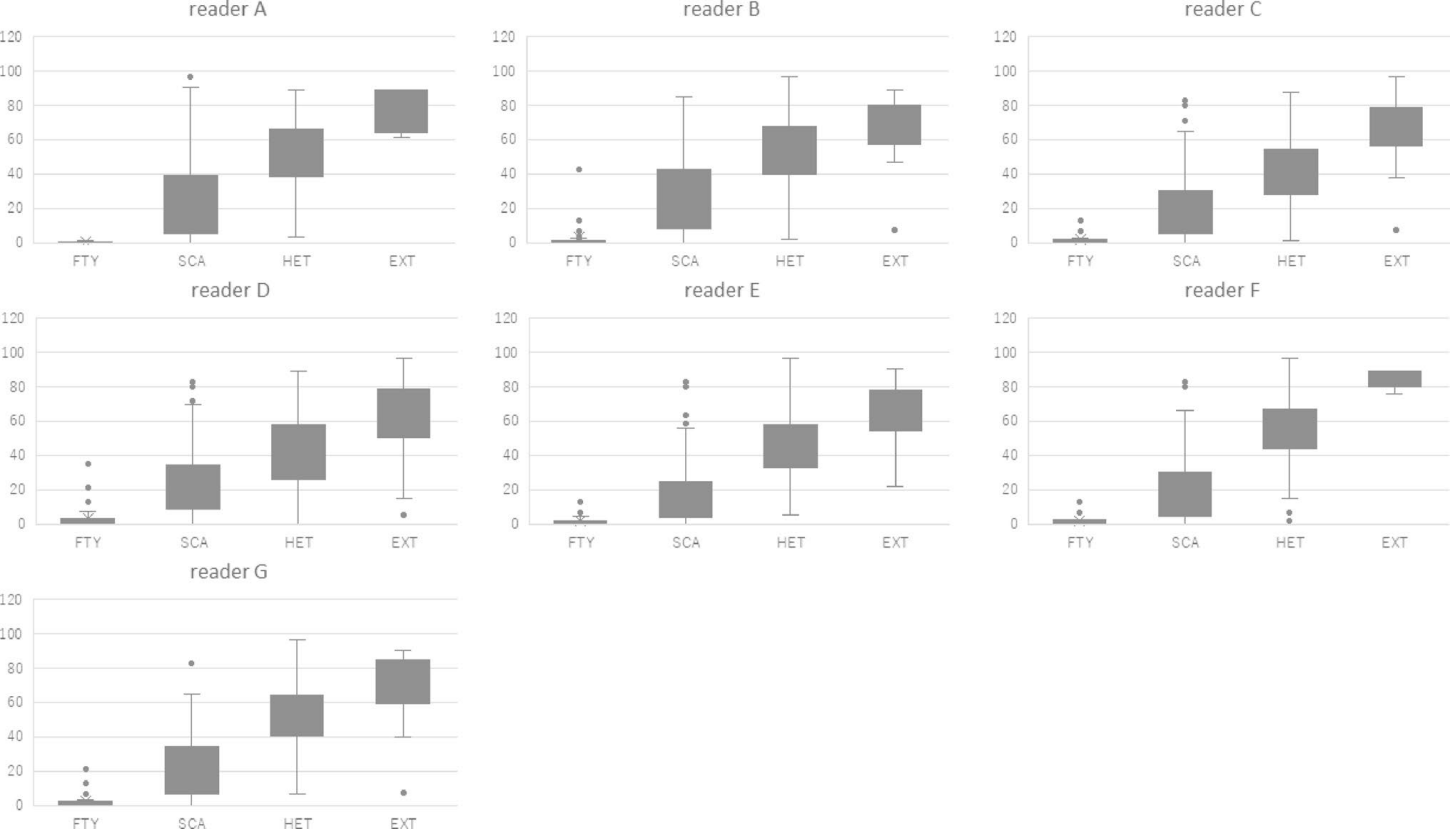
Plotted graph MDR distribution across visual breast composition categories by human readers

A total 300 mediolateral oblique (MLO) mammographic images of normal breast tissue were obtained at our hospital. Each image was manually annotated to delineate the breast gland tissue, the adipose tissue, and the pectoralis major muscle, producing corresponding masking images. The dataset was split into 240 cases for training and 60 cases for testing. All images were scaled down to 320 × 384 pixels and used to train a semantic segmentation model, based on the U-Net architecture [5]. U-Net is a deep learning encoder-decoder network that incorporates skip connections for precise pixel-wise classification. In our study, the U-Net was trained to predict the mask images directly from the original mammograms (Figs. 1a and 2). Once the pectoralis major region was identified by U-Net, we computed its average pixel intensity, denoted D_mj_, Within the breast glandular region (A_gl_) identified by the network, pixels with intensities higher than D_mj_ were classified as dense tissue (A_d_). The mammographic dense rate (MDR) was then defined as the ratio of A_d_ to the total glandular area A_gl_ (Figs. 1b, 3).

$$MDR=\frac{{{A_d}}}{{{A_{gl}}}}$$

**Fig. 4:**
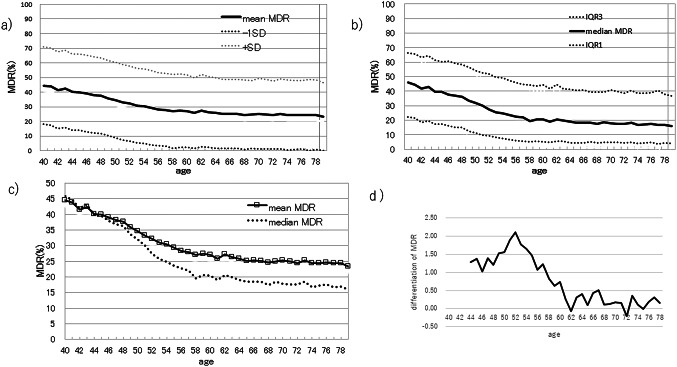
Mean MDR and median MDR **a**) Mean MDR for each age is plotted on a graph. **b**) Median MDR for each age is plotted on a graph, **c**) Age-related divergence between mean and median MDR, **d**) Moving average of temporal differentiation in MDR

In collaboration with the Yokohama City Medical Association, we collected mammographic images for 134,411 screening examinations performed between January 2019 and April 2022. After excluding tomosynthesis (3-D) studies, 240,465 MLO images remained, including both sides of breasts. These images comprise cases of primary breast cancer, benign lesions, post-operative breast, and breast-augmentation patients. For each image, the MDR was calculated and stratified into age cohorts ranging from 40 to 79 years. For each age, the mean, standard deviation, median, interquartile range 1 (25th percentile), and interquartile range 3 (75th percentile) of MDR were calculated and shown in Table 1. The mean MDR and the median MDR for each cohort were plotted to illustrate the age-related trend in breast density (Fig. 4a and b). For the mean MDR graph, the standard deviation was added. For the median MDR, interquartile range 1 and interquartile range 3 were added. The mean MDR and the median MDR for each age were plotted on a same graph so that the differences of changes between them can be visually recognized (Fig. 4c). In addition, the temporal differentiation in MDR using moving averages were shown in Fig. 4d.

The protocol for this study was approved both by the ethics committee of Shonan Memorial Hospital and the ethics committee of Yokohama City Medical Association. All data were anonymized and handled in accordance with privacy regulations. This research received no specific grant from any funding agency in the public, commercial, or not-for-profit sectors.

## Results

The AI model was trained and achieved a DICE coefficient of 0.967 (Fig. 2). Using this model, the MDR was automatically calculated for each image. Images and their corresponding MDR values were grouped by the patients’ age at the time of the mammograms. For each age, the number of the images and the associated MDR were shown in Table 1. The mean age was 56.6 ± 11.2 years. The mean number of images per age cohort was 6,011.6 ± 2,126.7 (range 1,949–14,106). Overall, the mean MDR was 31.7 ± 25.9% (range 23.3 − 44.5%), and the median MDR was 26.9% (range 16.0 − 45.9%).

**Table 1:**
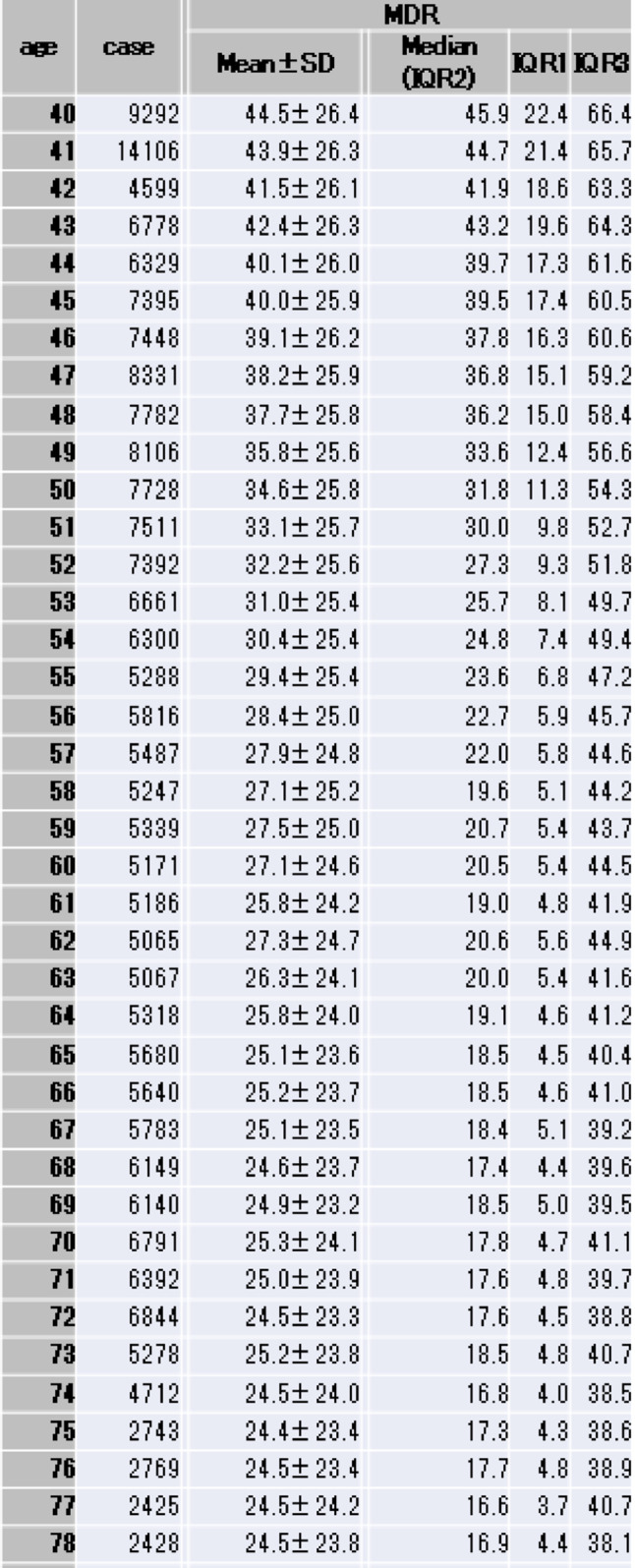
MDR

^*^SD standard deviation, *IQR* interquartile range

Seven human readers had evaluated breast compositions without utilizing AI model. The evaluations of breast composition highly related with the MDR (Fig. 3).

Figure 4 shows the mean (Fig. 4a) and median (Fig. 4b) MDR values plotted against age. In the early 40 s, the mean and median MDRs were similar, but the gap widened with increasing age (Fig. 4c). This discrepancy indicates that MDR does not decline uniformly across all individuals; some populations exhibit a pronounced age-related decline, while others maintain relatively high MDRs. Figure 4d shows the rate changes of MDR in moving average formula. The rate of changes dropped close to zero at age 62, representing the changes in MDR reaches plateau between the age of 58 and 62. This indicates the rate of changes in MDR appears to shift around the age of 60. In fact, median MDR decreases rapidly from 36.2% to 20.7% between the ages of 40 and 59, corresponding to a 25.2% reduction or 1.33% per year, whereas the decrease is less pronounced from 20.5% to 16.0% between the ages of 60 and 79, representing a 4.5% reduction or 0.24% per year.

**Fig. 5:**
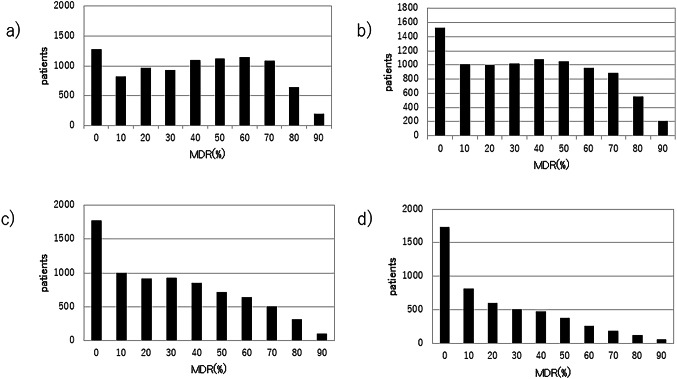
MDR distribution by age MDR distribution at the age of **a**) 40 years, **b**) 45 years, **c**) 50 years, **d**) 60 years

**Fig. 6:**
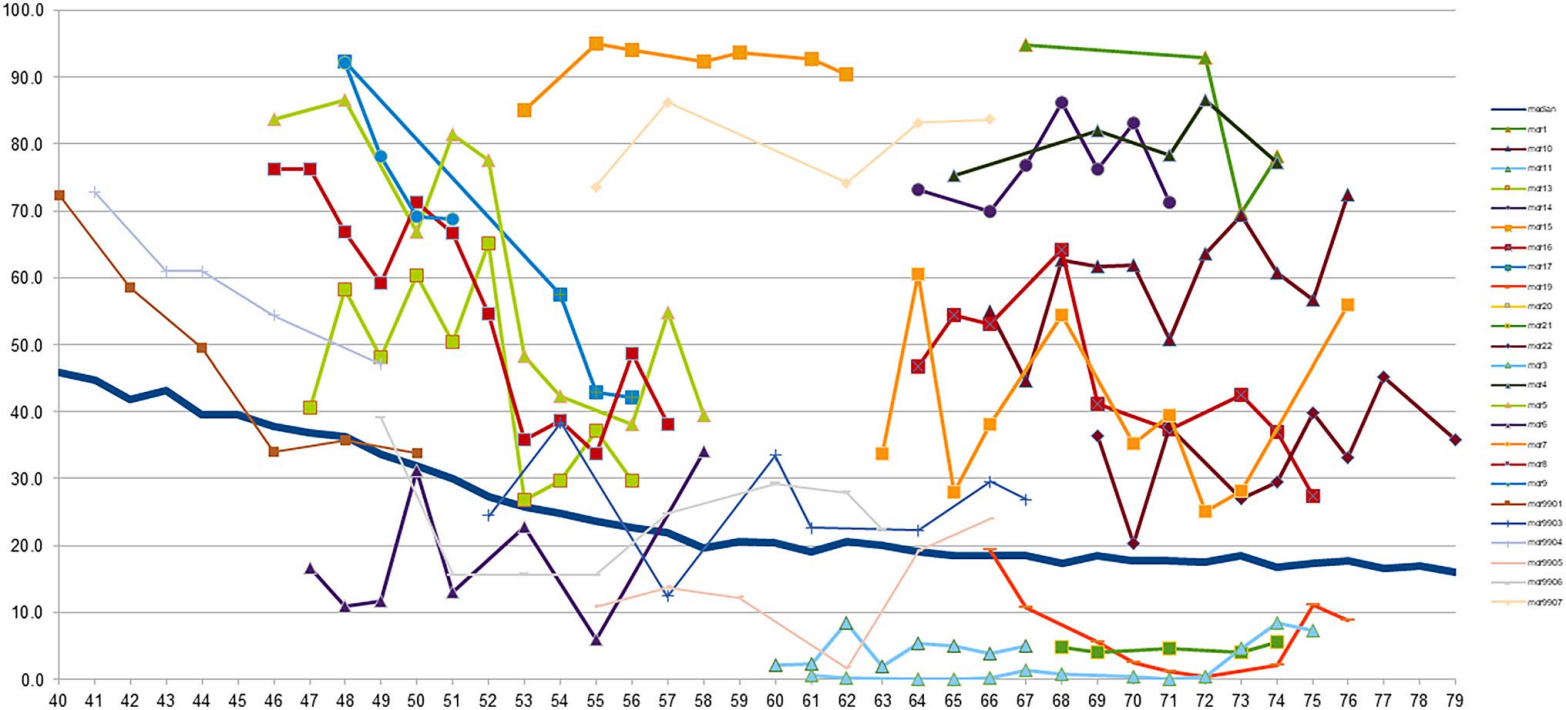
Individual MDR trajectory and median MDR The age-related trend of MDR for each individual

Figure 5 shows the MDR distributions for ages 40, 45, 50, and 60 (Fig. 5a, b, c and d correspondingly). The histograms show a significant shift during the premenopausal age. At age of 40, only 13.8% of women have MDR below 10%. In contrast, at the age of 60, 33.5% of women have MDR below 10%, while 19.8% of women still exhibit MDR greater than 80%. Figure 6 shows the age-related MDR trajectories for each individual examined. Most patients show a rapid decline in MDR during the perimenopausal years, but a subset maintain elevated densities throughout. These individual MDR variations demonstrates the existence of both groups: those whose MDR declines and those whose MDR remains unchanged.

Two illustrative cases are described below.

**Fig. 7:**
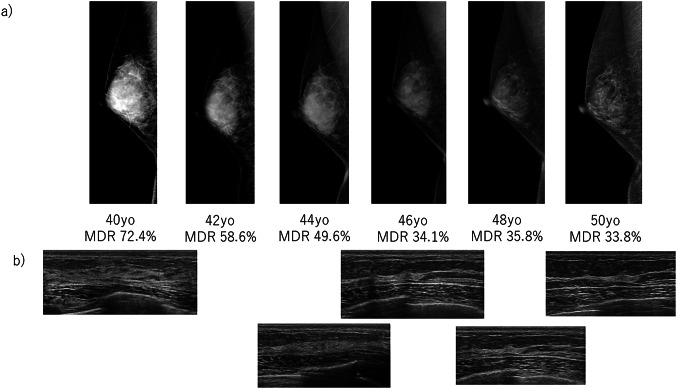
Case 1. a case in which MDR declined with age

Case 1: During the decade from age 40 to 50, the MDR dropped sharply. Correspondingly, mammograms revealed increasingly fatty breast tissue (Fig. 7a), and the ultrasound images confirmed the same trend with fattening and narrowing of the tissue (Fig. 7b).

**Fig. 8:**
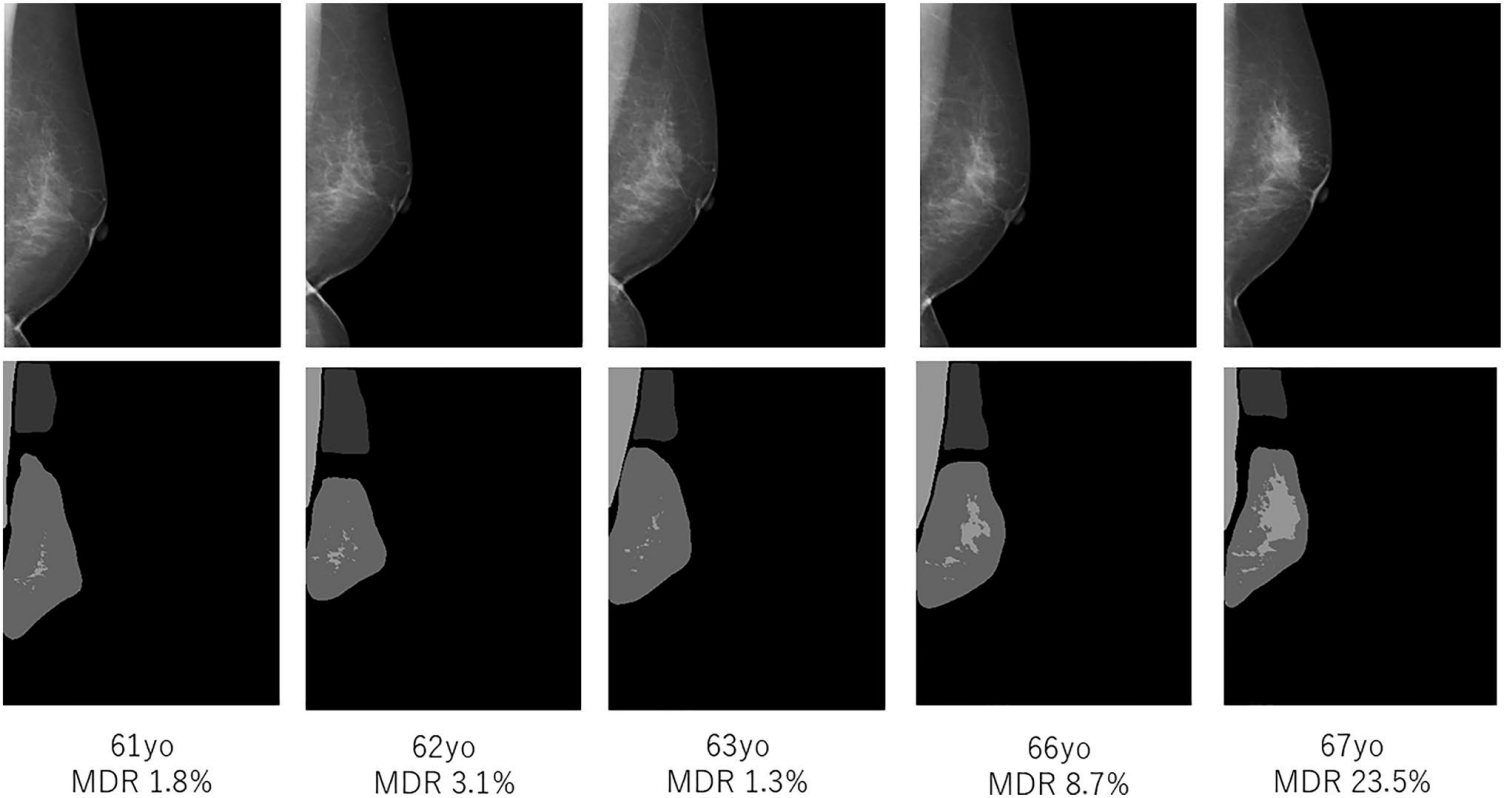
Case 2. a case of breast cancer whose MDR increased with aging

Case 2: A breast cancer was diagnosed at age 67; surgery revealed a non-invasive ductal carcinoma measuring 45 mm (Fig. 8). Longitudinal MDR measurements showed no significant change until age 63, after which MDR began to rise gradually. Heatmap analysis of the images demonstrated a progressive spread of density, suggesting that continuous MDR monitoring might serve as a tumor-maker for breast cancer detection.

## Discussion

Breast composition classification has evolved through several landmark methods. Wolfe first introduced the parenchymal pattern classification [6], followed by Tabar’s building-block approach [7] and Boyd’s percent-density method [8]. These early systems were subsequently refined and consolidated into the current BI-RADS 5th edition standard [2]. The primary aim of such classification is to estimate both the risk of developing breast cancer and the potential for tumor masking. However, visual grading suffers from significant inter-observer variability, rendering uniform assessment difficult. Astley reported that although the visual analogue scale (VAS) is useful cancer-risk predictor, the large inter-observer variability makes it impractical for population-based screening [9]. To overcome this limitation, researcher have pursued automated breast composition determination. The Cumulus system [10], an image-analysis-based semi-automatic software, has been adopted as the gold standard in Europe and the United States. Cumulus demonstrates a strong correlation with both breast density and breast-cancer risk, although it still required human intervention for ROI delineation. More recently, fully automated density-measurement tools such as Quantra [11] (Hologic) and Volpara [12] (Matakina Technology Limited) have been released. Volpara, in particular, shows a robust correlation with BI-RADS scores, and higher reliability and reproducibility than other assessment methods [13, 14]. Its use of raw, unprocessed mammograms, however, limits applicability when only processed images are stored. Its relatively high cost has also hindered widespread global adoption. Until recently, rule-based machine learning approaches to automated density assessment yielded limited accuracy. In contrast, AI-driven methods are now gaining traction. Rigaud reported that AI can automatically classify mammograms according to BI-RADs [15]. Li employed convolutional neural networks to extract and score breast tissue, yet many institutions cannot adopt these methods because it relies on unprocessed images [16]. Lehman employed a ResNet-based network to perform qualitative BI-RADs grading, and reported 77% agreement with human readers [17]. While this research demonstrates the feasibility of “human-like” evaluation, it does not resolve the core problem of breast density assessment. Numerous studies have reported that automated, quantitative density measurement is more accurate than purely qualitative classification [18]. FDA-approved AI applications such as IntelliMammo density a.i.™, PowerLook^®^ Density Assessment, and Volpara TruDensity^®^, provide volumetric density scores. However, each of these platforms has notable limitations: they require raw, unprocessed mammograms; archived images cannot be re-analyzed; and they are only available from specific vendors.

In Japan, the Breast Composition Judgment Atlas adopts four classifications, similar to BI-RADs categories. However, unlike BI-RADs, the dense-area percentage relative to the pixel intensity of the pectoralis major muscle is estimated by visual assessment. The denominator is the area in the breast that is expected to contain glandular tissue, while the numerator is the sum of all areas whose pixel values are equal to or exceed that of the pectoralis major muscle. The resulting percentage are interpreted as follows: less than 10% is defined as “fatty,” 10% to less than 50% as “scattered,” 50% to less than 80% as “heterogeneous,” and 80% or more as “extreme dense”. Heterogeneous and extreme dense classes are collectively termed as dense breasts. In terms of masking risk, Koyama [19] reported moderate inter-observer agreement (Fleiss’ κ = 0.553) in the breast-composition assessment, highlighting the lack of standardized criteria. This variability hampers the development of robust scientific evidence in Japan, underscoring the need for fully automated breast-composition measurement software.

In our study, the AI system performs fully automated calculation of the Mammographic Density Ratio (MDR), which can help standardize dense-breast evaluation. By utilizing a standardized software platform, meta-analysis of breast-cancer risk and masking risk become feasible, paving the way for nationwide evidence on breast cancer screening and risk assessment. Our finding shows that MDR declines steeply from age 40 through the late 50 s, and then tapers after age 60. The divergence between mean and median MDR across age cohorts indicates that some populations experience a pronounced decline in density while others maintain relatively high MDRs. Continuous MDR monitoring may therefore serve as a tumor marker, providing early detection of breast cancer.

## Limitations

Our study has several limitations. First, the AI model was trained on a relatively small dataset, which may compromise its robustness. Larger, more diverse training datasets are needed. Second, the ground-truth mask images for the various tissue regions were generated by a single annotator, so they do not represent as a true gold standard. Third, because the AI model had been trained on images from specific vendors, its segmentation performance may degrade when applied to scans from other machines. Based on these limitations, the current research is a pilot study, and large-scale additional studies are required in clinical practice. To enable widespread deployment, future work must develop a model trained on multi-institution mammograms with consensus-approved annotations, ensuring applicability across diverse clinical settings.

## Conclusion

Measuring the MDR permits the prediction of an individual’s future density trajectory. Consequently, MDR can be used to estimate cumulative breast cancer risk and the masking risk over time. For patients identified as high risk, these data support the implementation of personalized screening strategies, such as supplementing mammography with ultrasound or MRI, to improve detection rates. By tracking MDR, clinicians can forecast the age at which a patient’s breast density will fall below clinically relevant thresholds. During the window of elevated density, a combined screening protocol may be especially effective. Looking ahead, specialized organizations, such as the Japan Breast Cancer Screening Society and The Japan Central Organization on Quality Assurance of Breast Cancer Screening (JCOQABCS) will need to spearhead the validation, quality control, and ongoing maintenance of AI-based mammographic analysis to ensure its reliability and safety in clinical practice.
